# Phytochemical Constituents and Antioxidant Activity of *Oudneya Africana* L. Leaves Extracts: Evaluation Effects on Fatty Acids and Proteins Oxidation of Beef Burger during Refrigerated Storage

**DOI:** 10.3390/antiox8100442

**Published:** 2019-10-01

**Authors:** Hafedh Hajlaoui, Soumaya Arraouadi, Hedi Mighri, Mouna Chaaibia, Néji Gharsallah, Gaspar Ros, Gema Nieto, Adel Kadri

**Affiliations:** 1 Laboratory of Transmissible Diseases and Biologically Active Substances (LR99ES27), Department of Microbiology, Faculty of Pharmacy, University of Monastir, Avicenne Street 5000, Tunisia; bio.hafedh@gmail.com; 2 Research Unit Valorization and Optimization of Resource Exploitation (UR16ES04), Faculty of Science and Technology of Sidi Bouzid, University of Kairouan, Campus University Agricultural City—Sidi Bouzid 9100, Tunisia; 3 Regional Center of Agricultural Research (CRRA) Sidi Bouzid, Gafsa Road Km 5, PB 357, Sidi Bouzid 9100, Tunisia; bio.soumaya@gmail.com; 4 Range Ecology Laboratory, Arid Region Institute, University of Gabes, El-Jorf Road Km 22.5, Medenine 4119, Tunisia; mighrih@yahoo.fr; 5 Laboratory of Plant Biotechnology, Faculty of Science, University of Sfax, Soukra Road km 4, BP 802, Sfax 3038, Tunisia; chaaibia.mouna@hotmail.fr (M.C.); neji.gharsallah@fss.rnu.tn (N.G.); lukadel@yahoo.fr (A.K.); 6 Department of Food Technology, Food Science and Nutrition, Faculty of Veterinary Sciences, Regional Campus of International Excellence “Campus Mare Nostrum”, Espinardo, 30071 Murcia, Spain; gros@um.es; 7 College of Science and Arts in Baljurashi, Al Baha University, Al Baha, P.O.Box (1988), Saudi Arabia

**Keywords:** *Oudneya africana*, antioxidant activity, HPLC-MS phenolic analysis, beef burgers, TBARS, protein thiols groups, sensory analysis

## Abstract

Five *Oudneya Africana* (OA) leaves extracts were screened for their total phenolic (TPC), total flavonoid (TFC), condensed tannins (CTC) content, as well as their antioxidant capacity. The highest amount of TPC (661.66 ± 0.08 mg GAE/g), TFC (344.68 ± 0.44 mg QE/g) and TCT (90.18 ± 0.49 mg CE/g) was recorded to ethanol, acetone, and dichloromethane extracts, respectively. For 2,2-diphenyl-1-picrylhydrazyl (DPPH) (22.00 ± 0.03 µg/mL) and Reducing Power Assay (FRAP) (269.00 ± 0.01µg/mL) assays, ethanol extract showed the potent activity, while with ABTS test, acetone extract was the most active (761.15 ± 0.09 µg/mL). HPLC-MS analysis of acetonic and ethanolic extracts reveals the predominance of quinic acid, chlorogenic acid, 4-*O*-caffeoylquinic acid, and rutin compounds. The addition effect evaluation of OA extracts in beef burger preservation demonstrates the powerful effect (*p* < 0.05) of acetonic and ethanolic ones (0.03%) to inhibit lipids oxidation during storage for 10 days, given by the lowest increase in Thiobarbituric Acid-reactive Substances (TBARS) values as compared to the (−) control with a significant difference between free thiols values. In addition, these two extracts appear to be effective (*p* < 0.05) for pH stability, color, and sensory parameters as compared to (+) and (−) controls and aqueous extract. Hamburger odour was considered as a dependent variable in multiple linear regression analysis, where the models results showed that physicochemical parameters determine more burger odour than sensorial ones.

## 1. Introduction

Lipid and protein oxidations that occur in food products are one of the major concerns in food technology. They are responsible for rancid odors and flavours of the products, with a consequent decrease in nutritional quality and safety, caused by the formation of secondary toxic compounds [1]. Additionally, the presence and growth of pathogenic microorganisms (bacteria, fungi) in food may cause its spoilage, which alters its quality [2]. Indeed, for the food industry, antioxidants play a vital role in meat products by preventing the oxidative rancidity and retention of both color stability and shelf life [3]. Therefore, the meat industry is increasingly searching for natural solutions rather than synthetic additives [4,5], which were restricted due to their side effects, such as carcinogenesis [6,7,8]. Hence, the need for natural compensations has alarmingly increased nowadays. Thus, the development of effective natural antioxidants compounds has been investigated; more especially, phenolic substances, as part of plant extracts, have been largely precious in reducing or preventing lipid and protein oxidation in meat products by serving as radical scavengers by hydrogen atom or electron donators [9,10]. On the other hand, the damages that are caused by diseases where reactive oxygen species (ROS) are involved were reduced by the incorporation of these compounds in the human diet. In fact, the formation of ROS, such as superoxide ion (O_2_^−^), hydroxyl radical (OH^.^), and Hydrogen peroxide (H_2_O_2_), has often been reported to induce DNA damage, protein carbonylation, and lipid peroxidation, which causes a variety of chronic health disturbances and diseases [11,12]. These natural compounds are largely widespread, not only in aromatic and medicinal plants, but also are found in other plants that are bred in severe climatic conditions, such as Saharan plants. Later, these may be considered as a potential reservoir of bimolecular endowed with a higher capacity of antioxidant activities [13]. This richness of antioxidant compounds has a health promoting effects that attracted a big deal of scientific interest [14,15,16,17,18].

*Oudneya Africana* (OA) is an endemic Saharan plant of the Brassicaceae family mostly that is distributed in Libyan, Tunisian, Algerian, and Moroccan Sahara desert [19], it is known under the Arabic name “Alga” or “Hannet ibel”. The principal role of this plant in the desert is the stabilization of mobile dunes, potentially useful for rehabilitation of degraded ecosystems and food for goats and camelids [20,21], although it has also been used for its therapeutic properties in folk medicine in the form of infusion for the treatment of various lesions of the stomach, colon, liver, fevers, and scorpion bites [22,23,24]. Furthermore, this plant was used against skin illness [25]; however, leaves, stems, and seeds extract displayed important antioxidant, anti-inflammatory, and antimicrobial effects [26].

Taking into account the above facts, this study aimed to provide the phytochemical contents and elucidate the phenolic composition by HPLC-MS analysis as well as the antioxidant potential of OA leaves extracts. Additionally, the evaluation of the inhibition effect of three extracts on fatty acids and proteins oxidation of beef burger stored at 4 °C have been investigated. Finally, multiple linear regression analysis was used to select the powerful extract that is able to shelf life beef burger on the basis on hamburger odor (HO) sensory parameter. 

## 2. Material and Methods

### 2.1. Chemical and Reagents

Folin-Ciocalteu reagent, sodium carbonate anhydrous (Na_2_CO_3_), gallic acid, sodium nitrite solution (NaNO), sodiumhydroxyde (NaOH), aluminum chloride hexahydrate solution (AlCl_3_, 6H_2_O), iron (III) chloride anhydrous (FeCl_3_), and catechin were purchased from Fluka (Buchs, Switzerland). 2,2-diphenyl-1-picrylhydrazyl-(DPPH), 2,6-di-tert-butyl-4-hydroxytoluene (BHT), Trolox (6-hydroxy-2,5,7,8-tetramethylchroman-2-carboxylic acid), and 2,2’-azino-bis(3-ethylbenzothiazoline-6-sulphonate (ABTS) were purchased from Sigma-Aldrich (Murcia, spain), Tris (hydroxymethyl)-aminomethane (TRIS), and l-cysteine of analytical grade were obtained from Merck, Darmstadt, Germany. 5,5-dithiobis (2-nitrobenzoic acid) (DTNB) all of analytical grade were obtained from Fluka, Stenheim, Germany. Sodium dodecyl sulfate (SDS) was obtained from AppliChem GmbH, Darmstadt, Germany. Commercial mix was purchased from a supermarket (Murcia, spain).

### 2.2. Plant Material and Extraction

OA was collected from Douz (South of Tunisia) in April 2017. The leaves were rinsed with distilled aqueous immediately after harvest and then left to air dry in a sterile environment in shade and grounded to a fine powder. Preparation of solvent extracts was adapted from Matu and Staden [27] with modifications. Five solvents (hexane, dichloromethane, acetone, ethanol, and aqueous) were used. The extracts were obtained by maceration of 100 g of plant material with 600 mL of each solvent for 72 h. The filtrates were concentrated by rotary evaporator (BüchiRotavapor R-200 flawil, schweiz). The dry residue was then weighed and stored at 4 °C, and then submitted to all chemical and biological assays. Aliquots of extracts were filtered through a 0.22 µm membrane (Millipore Corporation, Bedford (Virginia, USA) and then injected in the HPLC-MS system.

### 2.3. Phytochemical Investigation

#### 2.3.1. Total Phenolic Content (TPC)

TPC was measured while using the Folin–Ciocalteu method as reported by Dewanto et al. [28] with few modifications. A mixture of 0.125 mL aliquot of 1 mg/mL sample extracts solution, 0.5 mL of distilled water and 0.125 mL of Folin–Ciocalteu reagent was carried out. After 3 min. of incubation, 1.25 mL of 7% Na_2_CO_3_ solution (w/v) and 1 mL of distilled water were added. This was followed by incubation for 90 min. at 23 °C in the dark, the absorbance was measured at 760 nm in a spectrophotometer apparatus (LABOMED, INC., Los Angeles, CA, USA) after incubation. A calibration curve was constructed using gallic acid standard solutions in the range 50–1000 µg/mL (*R*^2^ = 0.99). TPC was expressed as mg gallic acid equivalents per gram of dry extract (mg GAE/g DE).

#### 2.3.2. Total Flavonoids Content (TFC)

TFC were measured according to Dewanto et al. [28] with some modifications. An aliquot (0.25 mL) of 5 mg/mL extracts solution was added to 0.075 mL of 5% NaNO_2_ solution mixed and left standing for 6 min. before adding 0.15 mL of a freshly prepared 10% AlCl_3_ solution. After 5 min. of incubation at room temperature, 0.5 mL of 1 M NaOH solution was added. The final volume was adjusted to 2.5 mL with distilled water and thoroughly mixed. The absorbance of the mixture was measured at 510 nm in a spectrophotometer apparatus (LABOMED, INC., Los Angeles, CA, USA). A calibration curve was constructed using quercetin standard solutions in the range 50–500µg/mL. TFC were expressed as mg quercetin equivalents per gram of dry extract (mg QE/g DE).

#### 2.3.3. Condensed Tannins Content (CTC)

Tannins reactive to vanillin were analyzed by the vanillin method of Tomaino et al. [29]. Incubation at 25 °C of 1 mL of extract, placed in a test tube, and 2 mL of vanillin (1% in H_2_SO_4_ 7 M) in an ice bath was fulfilled. After 15 min., the absorbance of the solution was read at 500 nm. Concentrations were calculated as mg catechin equivalents per g dry extract from a calibration curve (mg CE/g DE).

#### 2.3.4. Analysis of Phenolic Compounds by HPLC-MS

The HPLC-MS method of phenolic compounds determination, as used in this study, has been developed by Mighri et al. [30]. The separation of phenolics was performed with a Shimadzu HPLC-MS 2020 system that was equipped with an online degasser (DGU-20A3R), a two binary pump (LC-20ADXR), an autosampler (SIL-20AXR), a column heater (CTO-20AC), and a diode array detector (SPD-M20A). Instrument control and data analysis was carried out while using Shimadzu lab solution V5.42 SP6 edition (Shimadzu, Kyoto, Japan) through Windows XP. The chromatographic separation was performed while using an AQUASIL C_18_ analytical column (150 mm × 3 mm × 3 μm particle size). Used as a stationary phase at 40 °C as temperature. The mobile phase consisted of methanol with formic Acid (0.1 mL/100 mL methanol) (solvent B) and water with formic Acid as solvent A (0.1 mL/100 mL water). The flow rate was kept at 0.4 mL/min. The gradient elution started with 90% A/10% B 0–45 min., 100% B 45–55 min., 90% A/10% B 55–55.1 min., 90% A/10% B 55.1–60 min. Photodiode array detector was set at 350 nm for acquiring chromatograms. The injection volume was 20 μL and the peaks were monitored at 250 nm. Peak identification was obtained by comparing the retention time and the UV spectra of the fraction phenolic chromatogram with those of pure standards, which were purchased from Sigma Aldrich and LGC standards.

Mass spectrometric analysis was performed on a Shimadzu mass spectrometer (Shimadzu, Kyoto, Japan). Mass spectra data were recorded on a negative ionization mode for a mass range of *m/z* 50–1500. Other mass spectrometer conditions were as follows: nebulizing gas pressure: 40 psi; drying gas flow: 12 L/min.; drying gas temperature: 400 °C; nebulizing gas flow: 1.5 L/min. The specific negative ionization modes (*m/z* [M−H]^−^) were used to analyze the compounds.

### 2.4. Antioxidant Assays

#### 2.4.1. Scavenging Ability on DPPH Radical

This activity was measured according to the method that was described by Hanato et al. [31], with slight modifications. Aliquots of 1 mL of the extract of each extracts were added to 0.25 mL of a DPPH methanolic solution. The mixture was shaken vigorously and then left to rest at room temperature for 30 min. in the dark. The absorbance of the resulting solution was then measured at 517 nm to determine the values that correspondedto the ability of the extracts to reduce the stable radical DPPH to the yellow-coloured diphenylpicrylhydrazine. Antiradical activity was expressed by 50% inhibition concentration values (IC_50_ µg/mL). A lower IC_50_ value corresponded to a higher antioxidant activity of plant extract. The scavenging ability of DPPH radical was calculated while using the following equation:

DPPH scavenging effect (%) = [(A_0_ − A_1_)/A_0_] × 100

where A_0_ refers to the absorbance of the control at 30 min., and A_1_ to the absorbance of the sample at 30 min. All of the samples were analyzed in triplicate.

#### 2.4.2. ABTS Scavenging Activity

ABTS was produced by the reaction between 5 mL of 7 mM ABTS solution and 5 mL of 2.45 mM potassium persulfate solution, and then stored in the dark for 16 h. The reaction mixture contained 950 µL of ABTS solution and 50 µL of each sample at various concentrations. The absorbance was recorded after 6 min. at 734 nm [32]. ABTS-scavenging ability was expressed as IC_50_ (mg/mL). All of the measurements were performed in triplicate.

#### 2.4.3. Reducing Power Assay (FRAP)

The FRAP was determined while using a slightly modified version of the method that was described by Oyaizu [33]. In brief, 250 μL of a sample at a concentration of 0.0625 to 1 mg/mL was mixed with a phosphate buffer (500 μL, 0.2 M, pH 6.6) and potassium ferricyanide (500 μL, 1%). Mixtures were then incubated in a water bath at 50 °C for 20 min. After adding 500 μL of trichloroacetic acid (10%) to each sample, all the mixtures were centrifuged at 1006×*g* for 10 min. Subsequently, 750 μL of the upper layer was mixed with 750 μL of distilled water and 50 μL of ferric chloride (0.1%) was added. The absorbance was measured at 700 nm and ascorbic acid was used as a positive control. A higher absorbance indicates a higher reducing power. The EC_50_ value (mg/mL) is the effective concentration, giving an absorbance of 0.5 for reducing power. All of the measurements were performed in triplicate.

### 2.5. Meat Conservation

#### 2.5.1. Preparation of Beef Burgers

Fresh beef meat shoulders were purchased from a local meat supplier. The meat was processed in a cool room at 4 °C. The preparation of beef burgers was prepared according to the Nieto method, with modifications [34]. Beef patties were prepared from three lots of meat batter (each 80 g) with 0.03% of acetonic extract, ethanolic extract and aqueous extract of OA. The fourth lot (80 g), without addition of extract, was used for preparation of negative control beef burgers. The fifth one was mixed with commercial mix (20 g/1000 g). This latter is used in a local meat industry for the elaboration of commercial burgers. The composition of commercial mix is vegetable fiber, salt, rice flour, meat protein, dextrose, corn starch, spices, preservative (E-221: sodium sulfite), antioxidant (E-301: sodium ascorbate, E-331: sodium citrate), and colorant (E-120: carminic acid). The total number of samples was 240 (four beef patties per lot × five different lots × four analysis days × three replicates each). The beef burgers were assigned to each day and packaged in polystyrene trays B5-37 (Aerpack) in BB4L bags (Cryovac) and then stored at 4 °C during 10 days. pH, color, sensory analysis, TBARS, and thiol were determined triplicate at days 0, 3, 7, and 10.

#### 2.5.2. Physicochemical Analysis

##### pH Value

5 g of sample was homogenized with 10 mL distilled water for 1 min. while using an Ultra Turrax^®^ (or with spatula)(IKA, La chapelle, France).The pH measurements were performed at Days 0, 3, 7, and 10.

##### Color

CIELAB color coordinates, L*, a*, and b*, were measured while using a hand held tristimulus Chroma Meter (CR-310 Minolta Camera Co. Ltd., Osaka, Japan), CIE standard ‘‘C” illuminant and 0-viewing angle geometry. Lightness, L*, chromaticity coordinates a* (redness/greenness), and b* (yellowness/blueness) were measured six times per meat sample and the respective averages were recorded.

##### Thiobarbituric Acid-reactive Substances (TBARS)

TBARS were measured to evaluate lipid oxidation on days 0, 3, 7, and 10 of storage at 4 °C, as described by Wang and Xiong [35]. The TBARS value, expressed as mg of malondialdehyde per kg of beef patties sample, was calculated while using the following equation:

TBARS = 9.48(A_532_/W_s_).

where A_532_ was absorbance at 532 nm, W_s_ was the beef patties sample weight (g), and 9.48 was a constant that was derived from the dilution factor and the molar extinction coefficient (1.52 × 10^3^ M^−1^ cm^−1^) of the red TBA reaction product.

##### Determination of Protein Thiol Groups: Derivatization with DTNB

Thiol concentration was determined spectrophotometrically after derivatization by Ellman’s reagent, DTNB [36]. In short, 2.0 g of meat was homogenized in 50 mL of 5.0% SDS that was dissolved in 100 mM TRIS buffer (pH 8.0) while using an Ultra Turrax. The homogenates were placed in an aqueous bath at 80 °C for 30 min., and then centrifuged for 20 min. at 3000 rpm (Biofuge Primo R, Axeb Laboratory products, Denmark). The supernatants were filtered (particle retention: 5–13 μm), and the protein concentration was spectrophotometrically determined at 280 nm while using a standard curve that was prepared from BSA. The filtrates were analyzed according to Liu and Xiong [37]. y mixing 500 μL sample, 2.00 mL of 100 mM TRIS buffer (pH 8.0), and 500 μL of 10 mM DTNB dissolved in 100 mM TRIS buffer (pH 8.0). The absorbance at 412 nm was measured before the addition of DTNB (ABS_412_-before) and after reaction with DTNB (ABS_412_-after) against a reference solution of 500 μL 5% SDS in 100 mM TRIS buffer (pH 8.0) and 2.50 mL of 100 mM TRIS buffer (pH 8.0). The mixture was allowed to react protected against light for exactly 30 min. A solution containing 2.00 mL 100 mM TRIS buffer (pH 8.0), 500 μL 5% SDS, and 500 μL 10 mM DTNB was used as blank sample (ABS_412_-blank).

Thus, after the corrected absorbance (ABS_412_-after − ABS_412_-before − ABS_412_-blank), the thiol concentration in the sample was determined by the calculation that was based on a five-point standard curve that ranged from 0.4 to 83.3 μM prepared from L-cysteine diluted in 5.0% SDS in 100 mM TRIS buffer (pH 8.0). The thiol content is given as the mean ± SD nmolthiol/mg protein of the samples collected from two independent beef burgers.

#### 2.5.3. Sensory Analysis

Sensory evaluation of all meat samples burgers elaborated with the three OA extracts, control burger, and burger elaborated with commercial mix, was assessed by a panel that consisted of eight panelists selected from students and staff of the laboratory. Each panelist was selected and trained according to ISO 8586-1 to carry out a quantitative descriptive sensory analysis [38]. In total, there were four training sessions, where different descriptors that were related to the odor and color of burgers were quantified and identified by the panelists. The descriptors used were: Hamburger odor (HO), rancid odor (RO), acid odor (AO), putrid odor (PO), extract odor (EO), meat color (MC), and fat color (FC). A linear scale of 1 (minimum) to 6 (maximum) was used: 1 = non-perceptible; 2 = very light; 3 = light; 4 = average; 5 = intense, 6 = very intense. The degree of spoilage and freshness of the beef burger are determined according to the scale mentioned above. For the sensory analysis, all of the evaluations were conducted in individual booths in a special room for sensory evaluation. In all sessions, the samples were coded with random three-digit numbers and were individually presented to the panelists. 

At the initial time of the experiment, and after 3, 6, and 9 days of preservation of meat samples at 4 °C, the panelists had to judge the acceptance of color and odor attributes of the raw meat samples. Sensory analysis was carried out according to ISO 4121 [39]. Each panelist evaluated four beef burgers from four different storage days. 

### 2.6. Statistical Analysis

A one-way analysis of variance (ANOVA) and Duncan’s post hoc-test was performed to determine significant differences between the treatments while using IBM SPSS Statistics 20. The means and standard errors were calculated. Differences among the mean values of the various treatments were determined by the least significant difference test. Stepwise multiple linear regression analysis were performed while using a Durbin–Watson statistic test, at 95% of confidence level, to determine the relationships, during storage at 4 °C of beef meat, between physicochemical change and sensory parameters. This analysis was used to assess the effects of treatments: Control (+) or (−), acetone, ethanol, and aqueous leaves extracts of OA.

## 3. Results and Discussion

### 3.1. Contents of Total Phenols, Flavonoids and Condensed Tannins of OA Leaves Extracts

The results of extraction yields (g/100 g dry extract) and the quantification of TPC, TFC, and CTC of the different OA leaves extracts are summarized in Table 1. 

As can be seen, the highest extraction yield was ascribed to aqueous extract, which is, respectively, 2.5, 5.86, 5.96, and 6.3 times higher than those of ethanol, acetone, hexane, and dichloromethane extracts. These results justify the richness of this plant on polar substances and they were related to the strongest solubility of phenolic compounds in the aqueous solvent. Significant differences (*p* < 0.05) in TPC, in TFC and in CTC for the five extracts were observed (Table 1). In addition, the TPC, TFC, and CTC of various AO leaves extracts ranged from 213.00 ± 1.41 to 661.66 ± 0.08 mg GAE/g, 3.97 ± 0.01 to 344.68 ± 0.44 mgQE/g, and 2.40 ± 0.02 to 90.18 ± 0.49 mgCE/g, respectively, with the highest phenolic level (661.66 ± 0.08 mg GAE/g) was allowed to ethanol, followed by acetone (496.16 ± 0.02 mg GAE/g) and dichloromethane (445.33 ± 3.27 mg GAE/g) extracts, respectively, while aqueous and hexane extracts exhibited the lowest polyphenolic contents. As regards to the flavonoids, acetone extract showed the highest concentration (344.68 ± 0.44 mg QE/g), followed by dichloromethane (301.61 ± 2.93mg QE/g), ethanol (296.28 ± 1.49 mg QE/g), and hexane (127.08 ± 0.73 mg QE/g) extracts, however the aqueous extract possess the lowest concentration (3.97 ± 0.01 mg QE/g). The effectiveness order in extraction of CTC was dichloromethane > hexane > acetone > ethanol > aqueous. Our results were much better than those obtained previously, which suggested a higher amount of polyphenols. It was found that TPC values were 108.6, 137.16, and 143.26 mg GAE/g and TFC values were 15.62, 21.96, and 27.63 mg QE/g for aqueous, hydro-alcoholic, and methanol extract, respectively [24]. Meanwhile, Khacheba et al. [40] working on OA collected from Laghou at town, in the steppe region of Algeria, demonstrate that leaves (15.86 ± 0.02 and 6.54 ± 0.03mg RE/g DW) aqueous extracts are richer in polyphenols and flavonoids than the pods (9.17 ± 0.04 and 3.61 ± 0.03 mg GAE/g DW). In comparison with our results, it can be noticed that polyphenols content not only depends on the solvent polarity, but also on geographical localities, harvest season, as well as plant age.

In this study, the poorly observed content in aqueous extract means that the major of flavonoids are not aqueous soluble. The weaker extractability effect of tannins is probably due to their higher molecular weight, which makes them to diffuse more slightly than oligomers in aqueous, leading to the formation of tannin-protein complexes [41].

### 3.2. Identification and Quantification of Phenolic Compounds by HPLC-MS

To the best of our knowledge, this is the first report that focused on the elucidation of polyphenol chemical composition by HPLC-MS of acetone and methanol OA extracts (chosen for their higher antioxidant activities) alone with their retention times, MS [M−H]^−^*m/z* and the amount of each compounds. As can be seen from the results that are disclosed in Table 2, twenty compounds were detected and identified, including phenolic acids, flavonoids, and tannins. A total of ten phenolic acids, eight flavonoids, and only two tannins were characterized in acetone extract, and nine phenolic acids, seven flavonoids, and one tannin were identified in ethanol extract, respectively. 

Concerning the acetone extract, six major compounds were identified, whose four phenolic acids are Chlorogenic acid (Rt = 12.06; *m/z* = 351.00), 4-*O*-caffeoylquinic acid (Rt = 13.013; *m/z* = 353.00), Caffeic acid (Rt = 13.301; *m/z* = 179.00), and 4,5-di-O-caffeoylquinic acid (Rt = 24.186; *m/z* = 515.00), one flavonoid (Rutin: Rt = 23.332; *m/z* = 609.00) and one tannin (Epichatechin: Rt = 14.781; *m/z* = 289.00). The same major compounds were identified of the ethanol extract, except tannin. The chromatographic profiles for the two extracts are indicated in the Supplementary Materials.

### 3.3. Antioxidant Activity of OA Extracts

The antioxidant potency of the different OA leaves extracts (hexane, dichloromethane, acetone, ethanol, and aqueous extracts) was assessed by DPPH, ABTS, and FRAP tests. As shown in Table 1, interestingly obtained DPPH scavenging data given by IC_50_ values in the range of 22.00 ± 0.03–343.02 ± 0.65 µg/mL were found with the significantly (*p* < 0.05) potent activity was obtained with ethanol extract (22.00 ± 0.03 µg/mL), which was five-fold higher than that of ascorbic acid (108.1 ± 0.06 µg/mL), followed by acetone (80.10 ± 0.03 µg/mL), aqueous (105.06 ± 0.65 µg/mL), and dichloromethane (343.02 ± 0.65 µg/mL) extracts. We also confirmed that the DPPH results were very much associated with the higher level of polyphenols (Table 1). In addition, HPLC-MS analysis of the ethanol extract (Table 2) showed an important richness in 4-*O*-caffeoylquinic acid (22,365.06 ± 1.04 µg/g of extract) and rutin (15447.12 ± 91.45 µg/g of extract), which are known by their role in biological activities. In fact, rutin is an important flavonoid in the pharmaceutical industry due to its pharmacological effects, such as anti-oxidative, anti-inflammatory, anti-diabetic, anti-adipogenic, neu-roprotective, and it was also involved in hormone therapy [42,43,44]. Besides, acetone extract also has important DPPH scavenging activity (80.10 ± 0.03 µg/mL), as well as having an approximately similar polyphenolic profile of ethanol extract (Table 2). Other results were reported for OA, indicating that methanol, aqueous, and hydroalcoholic extracts showed a very interesting DPPH radical scavenging with low values of IC_50_ (12.05, 14.91, and 24.57 μg/mL, respectively) [24].

For the ABTS assay, we note that acetone and ethanol extracts displayed the strongest scavenging inhibition than the other extracts (Table 1). Accordingly, the IC_50_ values ranged from 761.00 ± 0.09 µg/mL (acetone extract) to 2799.21 ± 0.01 µg/mL (hexane extract) and significantly differ (*p* < 0.05). The order of extracts ABTS•^+^ scavenging power was found to be as follows: ascorbic acid >acetone > ethanol > dichloromethane > aqueous > hexane extracts. The greatest ABTS•^+^ scavenging ability of acetone extract might be due to its higher flavonoic acids and flavonoids, especially 4-*O*-caffeoylquinic, chlorogenic, caffeic, 4,5-di-*O*-caffeoylquinic acids, and rutin as compared to other solvents.

Concerning the FRAP test, the results showed that ethanol, aqueous, and acetone extracts exhibit higher reducing power. Thus, the EC_50_ values are 269.00 ± 0.01, 805.00 ± 0.61, and 1006.12 ± 0.04 mg/mL, respectively, with a significant (*p* < 0.05) difference between the different extracts. Whereas, dichloromethane and hexane extracts showed relatively weaker activities (1423.16 ± 0.02 and 1627.12 ± 0.02 mg/mL, respectively) as compared to the ethanol ones. The highest antioxidant activity that was obtained with ethanol extract in this assay might be attributed to its richness in substantial ferric reducing activities, such as 4-*O*-caffeoylquinic acid and rutin, quinic acid, and chlorogenic acid.

### 3.4. Analyses of Meat Samples

Beef burgers samples that were elaborated with ethanol, acetone, and aqueous of OA leaves extracts (with 0.03% concentrations) have been the subject of physicochemical and sensory analyses while comparing them with negative (without natural extracts) and positive controls (with 2% of commercial mix). 

#### 3.4.1. Physicochemical Analysis

##### pH

Table 3 shows the effect of various OA extracts under investigation on the pH values of beef burgers stored at 4 °C between 0–10 days. 

On zero time, the pH of (+) control had the highest value (*p* < 0.05), followed by acetone, (−) control, aqueous, and ethanol. At the third day, a decrease in the pH was observed for control and all tested extracts except for ethanol. At the seventh and tenth day, a significant increase (*p* < 0.05) of pH was shown in all samples with the highest values recorded to aqueous extract and (−) control. Thus, after 10 days of storage, the obtained values are 7.15 ± 0.01 for aqueous extract and (−) control, 7.09 ± 0.01, 6.65 ± 0.01, and 6.64 ± 0.0 for acetone, ethanol extracts, and (+) control, respectively. According to statistical analysis, we could be able to discriminate between the various studied extracts, in their ability to maintain pH stability. In fact, after 10 days of storage, the ethanol extracts and mixed (+) control seems to be the most effective. These results can be explained on the one hand by ethanol extract richness in phenolic compounds (rutin, 4-*O*-caffeoylquinic acid, caffeic acid, and chlorogenic acid) and on the other hand by mixed composition (mainly preservative (E-221), antioxidant (E-301, E-331), and colorant (E-120)), which have antioxidant effects. The increase of pH values (*p* < 0.05) during the storage period might be attributed to the degree of meat spoilage. It is also due to protein alteration, resulting in the production of free amino acids, which leads to the formation of NH_3_ and amines compounds [45,46]. Other authors mentioned that fatty acids and their oxidation products in meat contribute to the increase in pH during storage [47,48]. In contrast; other studies show a decrease in the pH of the meat during refrigerated storage. In fact, [49] have shown that the pH values of the samples treatments decreased with storage time. The samples treated with *C. decapetala* leaves hydroalcolic extract (0.5%) had a lower pH value after storage (5.39). The changes in pH value during storage might be due to acidity produced by bacterial action on the muscle glucose and accumulation of the microbial metabolites due to bacterial spoilage in pork meat patties [50]. Generally, in the meat fat phase, hydroperoxides, which are a product of the primary oxidation, are unstable and rapidly transformed into a secondary product, such as acids, ketones, epoxides, or organic acids, and leads to pH changes [51,52].

##### Color

Color is one of the most relevant attributes with respect to the quality of meat products. Burger meat color parameter was measured by CIELAB coordinates (L*), (a*), and (b*) and the results are reported in Table 4.

Parameter L* values show few significant (*p* < 0.05) differences between all of the studied samples and for different storage periods. This does not allow for good discrimination concerning extracts effectiveness. However, the values of L* were ranged from 38.81 ± 0.39 to 45.10 ± 1.04 whatever sample nature and storage period are.

Redness (a*) is the most important color parameter for evaluating meat oxidation, which is due to the transformation of (bright red) oxymyoglobin to (brown) metmyoglobin. In this sense, decreases in a* values, turning to brown color makes the meat unacceptable to consumers [53,54]. In general, a* values significantly decrease (*p* < 0.05) with the increase of storage period for all samples. Furthermore, in each storage period, significant (*p* < 0.05) differences were shown between different samples of beef burgers. According to the a* value, reduction percentage acetone extract was the most efficient for maintaining beef burger redness (30.63%), followed by ethanol extract (33.33%) and (−) control (46.65%). Concerning aqueous extract and (+) control, the decrease of redness exceeds 50% (55.96 and 56.64%, respectively).

Regarding the yellowness (b*) value, significant (*p* < 0.05) differences were noted between different treatments. Except in aqueous extract and (+) control, the treatments samples explained a decrease of yellowness with increase of storage period. In fact, these latter treatments showed a stability of this parameter between 0 and 10 storage days. On the other hand, after 10 days of storage, samples that were treated by acetone and ethanol extracts showed a fewer decrease as compared to those non-treated which exhibited an important reduction of this coloration (43.5% of reduction).

Another study [55] regarding the addition of the natural antioxidant effect on Chorizo shelf-life showed that color parameters (L*, a*) were significantly (*p* < 0.05) affected, and those antioxidants were more effective in maintaining color. Furthermore, Chaytanya, K.V., et al. [12] focusing on active packaging containing natural extracts in beef patties, showed that film with 2% of *Persicaria minor* extract exhibited significant changes in the % metmyoglobin value, which indicated the minimum development of brown colour (*p* < 0.05).

According to the color parameters results, effectiveness to maintain color during storage period of same extracts, such as acetone and ethanol, was due to their ability to delay lipids oxidation and proteins alteration. On the other hand, this antioxidant was potentiality due to their richness of phenolic compounds, like rutin, 4-*O*-caffeoylquinic acid, caffeic acid, and chlorogenic acid. 

##### Measurement Thiobarbituric Reactive Species (TBARS)

The shelf-life of muscle foods is limited by an essential element, which is the deterioration in the particular lipid oxidation. The quantification of this latter is carried out with MDA compound [56,57]. Table 4 shows the effects of treatment on lipid oxidative. At day zero, statistical analysis showed that TBARS values were found to be the same for all beef burger samples, except those of (−) control. Over the storage period, the TBARS values were higher in (−) control samples than others in exception of aqueous extract, which they preserved their TBARS values (*p* > 0.05). Indeed, acetone, ethanol, and (+) control treatments had a significant (*p* < 0.05) decreasing effect on the TBARS values of beef burger samples. Furthermore, during storage time, there are significant differences between OA extracts and (−) control, which is due to their antioxidant properties. These results showed a good performance of OA leaves extracts and mix treatments ((+) control) to limit lipids oxidation during the refrigerated storage of beef burger. This performance could be attributed to the richness of phenolic contents in leaves (Table 2). These results are in agreement with literature data; in fact, [58] reported the efficiency of the essential oil of *M. piperita* to limit the lipids oxidation in minced beef samples to its chemical composition that shows a richness of menthol (33.59%) and menthone (33%). This latter, when used in combination with BacTN635, produced by *Lactobacillus* *plantarum* TN635 strain, has an interesting biopreservative effect on raw minced beef meat during refrigerated storage at 4 °C. In particular, the addition of *M. piperita* essential oils at 0.5% combined with BacTN635 at 1000 AU/g delayed the proliferation of spoilage microorganisms and limited lipid oxidation. In other studies, [49] showed that the ethanolic extract of *C. decapetala*, which is used in two concentration levels (0.1 and 0.5%), has a moderate lipid oxidation of beef patties during 11 days of refrigerated storage at 4 °C. The obtained TBARS values are significantly lower (*p* < 0.05) than the control. Thus, the concentration 0.5% showed a more limiting lipid oxidation than BHT treatment. This performing antioxidant activity was generally attributed to chemical composition.

##### Determination of Protein Thiol Groups

The free thiol, a sulfhydryl group (SH) of cysteine residue, is an important parameter that reflects the degree of protein oxidation in tissues. Protein oxidation is associated with a decrease in free thiol groups, by converting them into disulfides bridges that play an important role for evaluating oxidation protein modifications in meat [34,59,60]. 

In the present study, a significant (*p* < 0.05) decrease in free thiol, for all beef burger samples, was observed between 0 and 7 days (Table 4). However, no significant difference was shown between 7 and 10 days. In addition, after seven days of storage, no significant difference was noted between free thiol values of different samples. That is, because adding a 0.03% of OA leaves extract and 2% mixed formulation was ineffective in promoting discrimination between the treated samples and control. Contrarily, after 10 days of storage, a significant difference between the values of free thiol was recorded. Indeed, reduction percentage calculation of free thiol allowed for us to suggest that ethanol extract seems to be the most effective in limiting the oxidation of proteins than other extracts. However, the reduction percentages were 38.5, 61.56, 62.03, 64.6, and 65.65% for samples that were treated with ethanol extract, (+) control, acetone extract, aqueous extract, and (–) control, respectively. The preservation thiol group in meat during storage with an addition of natural products such as crude extract and essential oils has been widely studied by several authors [34,58,59,61]. However, this preservation depends on several factors, namely the dose of extract added, its chemical composition, and the conditions and the type of storage mostly in high-oxygen atmospheres [60]. In fact, Neito G., et al. [34], by studying the effect of rosemary essential oil, oregano, or garlic addition on protein oxidation in pork patties during storage under high-oxygen (MAP) or under aerobic conditions (AE), have shown that rosemary and oregano have an antioxidative effect on protein thiol loss, while garlic was found to be prooxidative, especially when applied in a high concentration. They added that progression of thiol loss differed between the packaging types, as a slightly more rapid thiol loss was noted for the control samples stored under MAP as compared to AE, which resulted in significantly (*p* < 0.05) different thiol levels for pork patties stored for three days in MAP or AE. On the other hand, different effects on the preservation of thiols group have been shown by both essential oils concentrations 0.05 and 0.4%.

Generally, several factors involved in meat quality degradation were essentially lipids and proteins oxidation, but we cannot exclude the microbial effect. In fact, microorganisms’ growth in meat during refrigerated storage, depends of nature and concentration plant extract. Studies of [62] showed that microbial population significantly decreased with the addition of *N. retusa* fruit extract and increased the concentration of this extract as compared to the control sample during nine days of refrigerated storage. Other studies focused on the addition of *Zizyphus jujube* [63] and *Phyllanthus emblica* [64] fruits extracts have been found to inhibit microbial proliferation in beef patties and in raw ground pork respectively.

#### 3.4.2. Sensory Analysis

The sensory analysis is of major importance for the introduction of new antioxidants in the meat industry, because it simulates consumer’s choice. Table 5 summarizes the effect of different extracts on the sensory properties of beef burgers during storage. 

Measured parameters were composed on five referred to odor (HO, RO, AO, PO, and EO) and two referred to color (MC and FC). In general, sensory analysis parameters fluctuate with the shelf life of the beef burgers. Indeed, in our analysis, a decrease of HO, EO, MC, and FC parameters and an increase of the RO, AO, and PO parameters with increasing storage time were shown. 

The acceptability of beef burger meat treated with 0.03% of different leaves extracts was acceptable until seven days (*p* < 0.05), but unacceptable at day 10 (*p* > 0.05). This fact was assessed by the scores of odor parameters (RO, AO, and PO), which were the highest (>3) at this date. Concerning color parameters (MC and FC), they decreased in scores and became the weakest and no discrimination between different extracts at 10 days of storage was observed.

The sensory spoilage of beef burger meat during storage was associated with a loss of color in both muscle and fat, a loss of the typical odor, and the appearance of slight rancidity. The MC and FC scores fell throughout storage, showing, the control samples a significant (*p* < 0.05) drop in this respect from day 3, accompanied by changes in the color coordinates, especially a* (Table 4). However, MC and FC in the different treatments showed no significant values (*p* > 0.05) during storage. From day 7 of storage, HO scores decreased in beef burger meat, but RO, AO, and PO showed an increase. 

These results agree with those for lipid and protein oxidation. The highest RO, AO, and PO scores corresponded to all samples with the greatest protein and lipid oxidation. In fact, these parameters showed significant and negative correlations (*p* < 0.05) with thiol groups (Supplementary Materials). In fact, in other work focused on the quality and sensory attributes of burger formulated from fresh beef cuts infused with citric acid and stored for eight days at 4 °C, sensory evaluation scores, showed a significant decrease for color, flavour, odor, texture, and overall acceptability, which ranged from 6.93–8.20 in the first day to 4.43–5.17 at the end of storage period [65].

#### 3.4.3. Correlation Matrix and Multiple Regression Analysis 

In our study we assumed to determine the degree of relationship that exists between physicochemical change and sensory measured variables. As an expression of relationship degree, the coefficient of correlation allowed us the subsequent interpretation of results. Among 78 correlations between measured parameters, 30, 36, 42, 48, and 52 are significant, respectively, in (−) control, aqueous, ethanol, (+) control and in acetone treatments. 

Comparisons between five matrices of inter parameters correlations in different conserved samples (Supplementary Materials) show that: (1) several correlations are specific in (−) control treatment, such as correlations between thiol and HO, PO and MC. These correlations showed that more beef burger proteins were altered more its odor was changed into a putrification (*r* = 0.606; *p* = 0.036), which was accompanied by losing beef burger meat color (*r* = −0.788; *p* = 0.002). (2) Specific correlations were also noted in treated samples. In (+) control, L* significantly and positively correlate with FC. This can be explained by the mix composition (vegetable fibre, salt, rice flour, meat protein, dextrose, corn starch, spices, preservative (E-221), antioxidant (E-301, E-331), colorant (E-120)) responsible for the modification of beef burger meat color in particular FC. In aqueous treatment thiol specifically presented a synergic relation with a*, EO and FC and an antagonist relationship was noted between L* and HO. Furthermore, two specific correlations were observed in ethanol treatment between pH and L*, TBARS, and thiol, and we also noted that HO has no correlation with all measured traits. In acetone treatment, two specific correlations were observed between b* and MC and HO and EO. 

On the other hand, in (−) control conditions no significant correlation was noted for L*, b* and EO with other measured traits. Comparatively with these conditions (30 significant correlations among 78 possible correlations), we registered more interactions when we add mix and different extract. PH parameter presented more significant correlation, with odor traits (RO, AO, and PO), in all of the treated samples. In fact, in other studies statistical analysis displayed that pH values of lamb burgers were significantly decreased by the addition of oregano extract, in comparison to sodium erythorbate [66].

We used multiple linear regressions as explaining models in order to select between extract, concerning its capacity to conserve beef meat. This showed more determination between these analyzed parameters. 

The main sensory system used by humans to sense flavour is olfaction; therefore, if the flavour of a particular substance is to be characterized, the use of smell can often provide us with suitable information [67]. Hamburger odor (HO) is considered to be important for several factors, including physical properties, such as color and appearance due to its importance in development and marketing of food product. However, in this section, the multiple linear regression analysis using the HO parameter as dependent variable was performed. Physicochemical values (pH, TBARS, thiol, a*, b*, and L*) and sensorial analysis (color and odor) were considered as independent variables. In fact, physicochemical and sensorial parameters was used for constructing regression models to predict overall acceptability in different studies in minced beef meat [58] and to determine whether the light backscatter response from fresh pork meat emulsions is correlated to final product stability indices [68].

##### Relationship between Hamburger Odor and Physicochemical Parameters.

The multiple linear regression analysis (Table 6) revealed a high correlation (*R*^2^ > 0.777) between HO and all parameters in (+) control, (−) control, acetone, and aqueous treatments. While, only 36% explain relationship between HO and b* variable (*R*^2^ = 0.360) in ethanol extract; more parameters are needed to perform determination coefficient. As shown in Table 6 the best single variable model contained the independent variable a* and the intercept. This model explained 81.6% of the variability observed in acetone extract. The HO was directly related to the increase of a*. In aqueous extract HO was significantly related to the increase of a* and to the decrease of L*. This model explained 87.5% of the observed variability. As shown in Table 6, in (+) control, TBARS had a positive effect on the HO, while in (−) control, TBARS and a* had a negative linear proportionality.

##### Relationship between Hamburger Odor and Sensory Parameters.

Multiple linear regression analysis (Table 6) revealed that determinant coefficients between HO and all the sensory parameters were less important than with physicochemical ones (0.518 < *R*^2^ < 0.792). No relationship has been determined between HO and sensory variables in ethanolic leaves extract treatment. Consumer choices for meat quality from a sensory point of view are influenced by appearance, tenderness, flavour, and succulence [69]. In our case, for the control and treated samples with aqueous leaves extract, the color had a positive effect on the predicted Hamburger odor. In fact, color was one of the most significant characteristics of meat, since it is the main element by which both fresh and cured meats were judged by a customer [70]. For samples that were treated by acetone leaves extract, the treatment significantly improved the odor (Table 6). This rancid odor possesses a positive impact on Hamburger odor (68.9%). The models that resulted from multiple linear regression analysis had shown that physicochemical parameters determine more hamburger quality odor in OA leaves extract.

## 4. Conclusions

Our results showed that dry leaves of OA harvested from southern Tunisia are rich in polyphenols. Ethanol extracts are the most rich in phenolic acids and flavonoids (Chlorogenic acid, 4-*O*-caffeoylquinic acid, Caffeic acid, 4,5-di-*O*-caffeoylquinic acid, and Rutin) as compared to other extracts. These extracts exhibited an important antioxidant activity and therefore can be considered as a promising source of natural antioxidant molecules used for the beef burger conservation. The addition of acetonic and ethanolic OA extracts (0.03%) seemed to be effective (*p* < 0.05) in limiting lipids oxidation of beef burger more than other extracts during storage at +4 °C for 10 days. These concentrations were ineffective in promoting discrimination between treated samples and control during 7 days of storage to limit proteins oxidation. Whereas, after 10 days of storage, a significant difference between values of free thiol was recorded. On the other hand, sensory analysis parameters showed a fluctuation with the shelf life of the beef burgers. A high correlation (R^2^ > 0.777) was determined between Hamburger odor and all physicochemical parameters in all treatments, except ethanol extract. Correlation with sensory parameters showed less determinant coefficients than the physicochemical ones (0.518 < *R*^2^ < 0.792). Generally, models that resulted from multiple linear regression analysis had shown that physicochemical parameters determine more Hamburger odor in OA leaves extract. All of these results support both the nutraceutical value and the benefits of new extracts of OA as a potential antioxidant agent to be used in the food industry. Industrialization and commercialization for OA benefits molecules is very promoted in Tunisia because of its large abundance in the Sahara region.

## Figures and Tables

**Table 1:** Extraction yield (%), total polyphenolic (TPC mgGAE/g), total flavonoid (TFC mgQE/g) and condensed tannins contents (CTC mgCE/g), DPPH radical-scavenging activity (IC_50_ µg/mL)), 2,2’-azino-bis(3-ethylbenzothiazoline-6-sulphonate (ABTS) (IC_50_ µg/mL) and Reducing Power Assay (FRAP) (CE_50_ µg/mL) of different extracts of *Oudneya Africana* leaves.

	Hexane	Dichloromethane	Acetone	Ethanol	Aqueous	Ascorbic Acid
**Yield** (%)	2.26	2.14	2.30	5.39	13.49	-
**TPC**	213.00 ± 1.41 ^e^	445.33 ± 3.27 ^c^	496.16 ± 0.02 ^b^	661.66 ± 0.08 ^a^	291.5 ± 0.81 ^d^	-
**TFC**	127.08 ± 0.73 ^d^	301.61 ± 2.93 ^b^	344.68 ± 0.44 ^a^	296.28 ± 1.49 ^c^	3.97 ± 0.01 ^e^	-
**CTC**	70.47 ± 1.41 ^b^	90.18 ± 0.49 ^a^	45.62 ± 0.01 ^c^	36.22 ± 0.03 ^d^	2.40 ± 0.02 ^e^	-
**DPPH**	-	343.02 ± 0.01 ^a^	80.10 ± 0.03 ^c^	22.00 ± 0.03 ^d^	105.06 ± 0.65 ^b,c^	108.1 ± 0.06 ^b^
**ABTS**	2799.21 ± 0.01 ^a^	1354.50 ± 0.06 ^c^	761.15 ± 0.09 ^e^	1075.25 ± 0.06 ^d^	1761.10 ± 0.03 ^b^	71.0 ± 0.04 ^f^
**FRAP**	1627.12 ± 0.02 ^a^	1423.16 ± 0.02 ^b^	1006.12 ± 0.04 ^c^	269.00 ± 0.01 ^e^	805.00 ± 0.61 ^d^	31.02 ± 0.48 ^f^

Each data point represents mean ± SD of three independent replicate. ^a, b, c, d, e, f^: Means followed by the same letters are not significantly different at *p* = 0.05 based on Duncan’s multiple range test.

**Table 2:** Identified by HPLC-MS analysis of acetone and ethanol extract obtained from *Oudneya Africana* Leaves.

Peak	Retention Time (min)	MS [M−H]^−^m/z	Compounds	Quantity in µg/g Extract	
				Acetone Extract	Ethanol Extract
**1**	2.255	191.00	Quinic acid	228.61 ± 7.41	1097.96 ± 50.48
**2**	4.768	169.00	Gallic acid	72.045 ± 2.06	17.98 ± 0.15
**3**	7.396	153.00	protocatchuic acid	242.98 ± 0.86	24.58 ± 0.10
**4**	9.583	289.00	Catechin (+)	17.521 ± 0.08	3.79 ± 0.18
**5**	12.060	351.00	Chlorogenic acid	459.5 ± 20.94	655.21 ± 43.88
**6**	14.781	289.00	Epicatechin	458.84 ± 13.35	-
**7**	13.013	353.00	4-*O*-caffeoylquinicacid	15,443 ± 10.47	22,365.06 ± 1.04
**8**	13.301	179.00	Caffeic acid	320.71 ± 2.53	69.38 ± 1.59
**9**	17.548	163.00	p-coumaric acid	192 ± 0.32	24.14 ± 0.15
**10**	21.868	579.00	Naringin	79.657 ± 3.12	-
**11**	21.978	359.00	Rosmarinic acid	55.898 ± 1.47	-
**12**	22.507	463.00	Hyperoside (quercetin-3-*O-*galactoside)	206.31 ± 5.39	56.11 ± 1.90
**13**	23.332	609.00	Rutin	18,635 ± 472.13	15,447.12 ± 91.45
**14**	23.564	717.00	Salvianolic acid	230.82 ± 7.97	88.44 ± 1.14
**15**	24.186	515.00	4,5-di-*O*-caffeoylquinic acid	557.08 ± 5.11	490.89 ± 9.36
**16**	25.551	447.00	Quercetrin (quercetin-3-*O-*rhamonoside)	44.976 ± 0.77	7.53 ± 0.73
**17**	27.075	271.00	Naringenin	2.757 ± 0.04	0.73 ± 0.10
**18**	28.802	481.00	Silymarin	13.998 ± 0.27	2.92 ± 0.23
**19**	32.536	329.00	Cirsiliol	38.613 ± 0.42	6.21 ± 0.10
**20**	37.233	283.00	Acacetin	125.19 ± 2.40	118.02 ± 7.99

**Table 3:** Average pH value (pH) in beef burgers added *Oudneya africana* leaves extracts (with 0.03%) stored at 4 °C during 10 days under simulated retail display conditions.

Extracts	Storage Day			
	Day0	Day3	Day7	Day10
**Acetone**	5.64 ± 0.01 ^b,C^	5.54 ± 0.03 ^b,D^	6.65 ± 0.54 ^c,B^	7.09 ± 0.01 ^b,A^
**Ethanol**	5.45 ± 0.02 ^d,D^	5.47 ± 0.01 ^c,C^	6.62 ± 0.46 ^d,B^	6.65 ± 0.01 ^c,A^
**Aqueous**	5.59 ± 0.01 ^c,C^	5.54 ± 0.02 ^b,D^	6.85 ± 0.26 ^a,B^	7.15 ± 0.01 ^a,A^
**(–)Control**	5.63 ± 0.01 ^b,C^	5.57 ± 0.01 ^b,D^	6.71 ± 0.41 ^b,B^	7.15 ± 0.01 ^a,A^
**(+)Control**	5.73 ± 0.01 ^a,C^	5.75 ± 0.02 ^a,C^	6.51 ± 0.14 ^e,B^	6.64 ± 0.01 ^c,A^

Each data point represents mean ± SD of three independent replicates. (^a,b,c,d,e, A,B,C,D^): Means followed by the same letters are not significantly different at *p* = 0.05 based on Duncan’s multiple range test. Small letters are used to compare means between extracts for each storage day, while capital letters are used to compare means between storage days for the same extract.

**Table 4:** Effect of *Oudneya africana* leaves extracts (0.03%) and commercial mix (2%) on color (L*, a* and b*), lipid oxidation (TBARS expressed in mg MDA/kg samples) and protein thiol loss (expressed in nmol/mg protein) in beef burgers stored at 4 °C during 10 days.

Extracts	Treatment	Storage Days			
		Day 0	Day 3	Day 7	Day 10
	L*				
**Acetone**		42.41 ± 1.02 ^aA^	41.53 ± 0.55 ^bB^	41.95 ± 1.18 ^abB^	42.31 ± 0.42 ^abA^
**Ethanol**		42.28 ± 0.52 ^aB^	44.22 ± 0.48 ^abA^	39.85 ± 0.59 ^bcC^	38.81 ± 0.39 ^cD^
**Aqueous**		39.51 ± 1.30 ^bBC^	42.91 ± 0.47 ^abA^	39.58 ± 0.85 ^cC^	40.24 ± 0.27 ^bcB^
**(–)** **Control**		43.10 ± 0.79 ^aB^	45.10 ± 1.04 ^aA^	43.02 ± 0.97 ^abB^	43.15 ± 0.59 ^aB^
**(+)** **Control**		44.84 ± 0.69 ^aA^	44.26 ± 0.55 ^abA^	43.77 ± 1.11 ^aA^	43.62 ± 1.03 ^aA^
	*a				
**Acetone**		12.11 ± 0.44 ^dA^	9.81 ± 0.13 ^dB^	8.99 ± 1.03 ^cB^	8.4 ± 0.32 ^bC^
**Ethanol**		15.72 ± 0.53 ^cA^	11.74 ± 0.36 ^bcB^	11.16 ± 0.43 ^abcBC^	10.84 ± 0.28 ^aC^
**Aqueous**		16.6 ± 0.68 ^cA^	12.88 ± 0.16 ^bB^	8.72 ± 1.60b ^cC^	7.31 ± 0.33 ^bD^
**(–)** **Control**		19.16 ± 0.52 ^bA^	10.32 ± 0.19 ^cC^	11.55 ± 0.38 ^bB^	10.22 ± 0.38 ^aC^
**(+)** **Control**		24.91 ± 0.34 ^aA^	16.88 ± 0.67 ^aB^	12.77 ± 0.33 ^aC^	10.8 ± 0.33 ^aD^
	b*				
**Acetone**		11.01 ± 0.20 ^bcA^	8.74 ± 0.28 ^bC^	8.68 ± 0.24 ^bC^	9.15 ± 0.21 ^bB^
**Ethanol**		11.95 ± 0.40 ^bA^	10.12 ± 0.24 ^aB^	9.07 ± 0.25 ^abC^	8.92 ± 0.25 ^bcC^
**Aqueous**		9.94 ± 0.40 ^cA^	9.34 ± 0.28 ^abA^	9.28 ± 0.04a ^bA^	9.46 ± 0.30 ^bA^
**(–)** **Control**		14.07 ± 0.18 ^aA^	9.82 ± 0.36 ^abB^	7.36 ± 0.23 ^cC^	7.94 ± 0.25 ^cC^
**(+)** **Control**		10.83 ± 0.37 ^bcA^	9.03 ± 0.25 ^abC^	9.77 ± 0.19 ^aB^	10.67 ± 0.27 ^aA^
	TBARS				
**Acetone**		0.54 ± 0.03 ^bA^	0.49 ± 0.02 ^bB^	0.47 ± 0.02 ^bBC^	0.44 ± 0.01 ^bC^
**Ethanol**		0.58 ± 0.03 ^bA^	0.56 ± 0.02 ^bA^	0.46 ± 0.01 ^bB^	0.45 ± 0.02 ^bB^
**Aqueous**		0.61 ± 0.09 ^bA^	0.46 ± 0.03 ^bA^	0.58 ± 0.12 ^bA^	0.54 ± 0.08 ^bA^
**(–)** **Control**		1.33 ± 0.32 ^aC^	1.98 ± 0.19 ^aB^	2.88 ± 0.28 ^aA^	2.95 ± 0.19 ^aA^
**(+)** **Control**		0.58 ± 0.03 ^bA^	0.48 ± 0.0 ^bB^	0.42 ± 0.00 ^bC^	0.40 ± 0.01 ^bC^
	THIOLS				
**Acetone**		40.96 ± 0.99 ^bA^	ND	19.36 ± 7.91 ^aB^	15.55 ± 1.44 ^cB^
**Ethanol**		32.60 ± 5.42 ^cA^	ND	20.74 ± 3.58 ^aB^	20.38 ± 0.62 ^abB^
**Aqueous**		48.14 ± 2.51 ^bA^	ND	24.56 ± 7.66 ^aB^	17.04 ± 2.28 ^bcB^
**(–)** **Control**		47.90 ± 4.61 ^bA^	ND	25.65 ± 11.34 ^aB^	16.45 ± 2.89 ^cB^
**(+)** **Control**		59.38 ± 6.77 ^aA^	ND	29.83 ± 3.65 ^aB^	22.82 ± 1.66 ^aB^

Each data point represents mean ± SD of three independent replicates (^a,b,c,d,A, B, C, D^): Means followed by the same letters are not significantly different at *p* = 0.05 based on Duncan’ multiple range test. Small letters are used to compare means between extracts for each storage day, while capital letters are used to compare means between storage days for the same extract. ND: not determined.

**Table 5:** Scores for in beef burgers added *Oudneya africana* leaves extracts (0.03%) and commercial mixed (2%) stored at 4 °C during 10 days.

Parameters	Extracts	Storage days			
		Day 0	Day 3	Day 7	Day 10
	Acetone	3.00 ± 1.29 ^bA^	2.57 ± 0.78 ^bA^	2.00 ± 0.81 ^abAB^	1.14 ± 0.37 ^aB^
	Ethanol	3.71 ± 1.79 ^abA^	2.57 ± 1.39 ^bAB^	2.28 ± 1.88 ^abAB^	1.14 ± 0. ^37aB^
**HO**	Aqueous	4.71 ± 1.38 ^abA^	1.42 ± 0.78 ^cC^	3.14 ± 1.77 ^aB^	1.14 ± 0.37 ^aC^
	(–)Control	4.14 ± 1.34 ^abA^	3.14 ± 0.89 ^abB^	1.42 ± 0.53 ^bC^	1.14 ± 0.37 ^aC^
	(+)Control	5.00 ± 1.41 ^aA^	4.14 ± 0.69 ^abA^	1.14 ± 0.37 ^bB^	1.14 ± 0.37 ^aB^
	Acetone	1.00 ± 0.00 ^aB^	1.57 ± 1.13 ^aB^	2.14 ± 1.21 ^bB^	3.28 ± 1.70 ^aA^
	Ethanol	1.00 ± 0.00 ^aB^	1.71 ± 1.11 ^aB^	1.85 ± 1.06 ^bB^	3.71 ± 2.05 ^aA^
**RO**	Aqueous	1.00 ± 0.00 ^aB^	1.14 ± 0.37 ^aB^	1.71 ± 1.25 ^bB^	3.85 ± 1.95 ^aA^
	(–)Control	1.00 ± 0.00 ^aB^	2.14 ± 1.06 ^aB^	2.42 ± 1.39 ^bB^	4.57 ± 1.81 ^aA^
	(+)Control	1.00 ± 0.00 ^aB^	1.28 ± 0.48 ^aB^	4.14 ± 1.06 ^aA^	4.71 ± 1.11 ^aA^
	Acetone	1.00 ± 0.00 ^aC^	1.85 ± 1.21 ^aBC^	2.00 ± 1.00 ^abB^	4.15 ± 2.00 ^aA^
	Ethanol	1.00 ± 0.00 ^aC^	1.14 ± 0.37 ^aBC^	2.00 ± 1.00 ^abB^	3.85 ± 1.86 ^aA^
**AO**	Aqueous	1.14 ± 0.37 ^aB^	1.14 ± 0.37 ^aB^	2.00 ± 1.00 ^bB^	3.71 ± 1.88 ^aA^
	(–)Control	1.00 ± 0.00 ^aC^	1.57 ± 0.53 ^aBC^	3.00 ± 1.00 ^abB^	4.57 ± 1.71 ^aA^
	(+)Control	1.00 ± 0.00 ^aB^	1.57 ± 0.53 ^aB^	4.00 ± 1.00 ^aA^	4.57 ± 0.97 ^aA^
	Acetone	1.00 ± 0.00 ^aB^	1.42 ± 0.78 ^aB^	1.71 ± 0.75 ^bB^	3.71 ± 1.79 ^aA^
	Ethanol	1.00 ± 0.00 ^aB^	1.00 ± 0.00 ^aB^	1.85 ± 0.89 ^bB^	3.42 ± 1.90 ^aA^
**PO**	Aqueous	1.00 ± 0.00 ^aB^	1.14 ± 0.37 ^aB^	1.71 ± 1.49 ^bB^	3.85 ± 1.57 ^aA^
	(–)Control	1.00 ± 0.00 ^aB^	1.57 ± 0.53 ^aB^	2.28 ± 1.38 ^bB^	4.57 ± 1.71 ^aA^
	(+)Control	1.00 ± 0.00 ^aB^	1.42 ± 0.53 ^aB^	4.42 ± 0.78 ^aA^	4.85 ± 1.06 ^aA^
	Acetone	3.57 ± 0.97 ^baA^	2.57 ± 0.97 ^bA^	2.85 ± 1.06 ^aA^	1.28 ± 0.48 ^aB^
	Ethanol	3.85 ± 1.46 ^aAB^	5.00 ± 1.00 ^aA^	3.28 ± 1.49 ^aBC^	2.00 ± 1.41 ^aC^
**EO**	Aqueous	4.28 ± 1.70 ^aA^	4.57 ± 1.51 ^aA^	1.57 ± 0.53 ^bB^	1.42 ± 0.78 ^aB^
	(–)Control	1.28 ± 0.48 ^bA^	1.42 ± 0.53 ^cA^	1.42 ± 0.78 ^bA^	2.00 ± 1.91 ^aA^
	(+)Control	3.14 ± 1.67 ^aA^	1.28 ± 0.48 ^cB^	1.28 ± 0.75 ^bB^	2.00 ± 1.82 ^aAB^
	Acetone	5.57 ± 0.53 ^abA^	3.28 ± 0.95 ^aBC^	3.57 ± 0.78 ^abB^	2.42 ± 1.39 ^aC^
	Ethanol	5.14 ± 1.06 ^abA^	3.57 ± 1.13 ^aB^	3.71 ± 0.95 ^aB^	3.14 ± 1.46 ^aB^
**MC**	Aqueous	6.00 ± 0.00 ^aA^	3.28 ± 0.95 ^aB^	2.57 ± 1.13 ^abBC^	2.00 ± 1.00 ^aC^
	(–)Control	5.14 ± 1.21 ^abA^	2.85 ± 0.69 ^aB^	3.28 ± 1.70 ^abB^	2.28 ± 1.11 ^aB^
	(+)Control	4.71 ± 1.25 ^bA^	3.57 ± 0.78 ^aB^	2.28 ± 0.75 ^bC^	1.71 ± 0.95 ^aC^
	Acetone	5.28 ± 0.95 ^aA^	4.28 ± 0.48 ^aA^	2.85 ± 1.46 ^aB^	2.42 ± 1.13 ^aB^
	Ethanol	5.57 ± 0.78 ^aA^	4.57 ± 0.78 ^aAB^	3.57 ± 0.97 ^aBC^	3.14 ± 1.34 ^aC^
**FC**	Aqueous	6.00 ± 0.00 ^aA^	4.71 ± 0.95 ^aB^	3.57 ± 1.27 ^aBC^	2.57 ± 1.39 ^aC^
	(–)Control	5.14 ± 1.46 ^aA^	4.28 ± 0.95 ^aAB^	3.71 ± 1.38 ^aAB^	2.71 ± 1.60 ^aB^
	(+)Control	5.00 ± 1.52 ^aA^	4.00 ± 1.00 ^aAB^	2.71 ± 1.38 ^aB^	2.57 ± 1.39 ^aB^

Each data point represents mean ± SD of three independent replicates. Scoring scale: (1: minimum; 6: maximum). HO: hamburger meat odour; RO: rancid odour; AO: acid odour; PO: putrid odour; EO: extract odour; MC: meat color; FC: fat color. (^a,b,c,A,B,C^): Means followed by the same letters are not significantly different at *p* = 0.05 based on Duncan’s multiple range test. Small letters are used to compare means between extracts for each storage day, while capital letters are used to compare means between storage days for the same extract.

**Table 6:** Stepwise multiple linear regression equations of Hamburger Oder (HO) versus physico-chemical and sensorial parameters of beef burger meat stored at 4 °C.

Parameters	Extracts	Regression Equation	R^2^
**Physico-chemical**	Acetone	HO = −4.208 + 0.903 a*	0.816
	Ethanol	HO = −0.780 + 0.600 b*	0.360
	Aqueous	HO = 26.546+ 0.752 a* − 0.692 L*	0.875
	(+)Control	HO = −1.123 + 0.881 TBARS	0.777
	(−)Control	HO = 9.927 - 1.210 TBARS − 0.493 a*	0.814
**Sensorial**	Acetone	HO = −4.184 + 0.830 RO	0.689
	Ethanol	-	
	Aqueous	HO = −0.254+ 0.805 MC	0.648
	(+)Control	HO = 3.605+ 0.534 MC − 0.524 PO	0.792
	(−)Control	HO = 1.184 +0.720 MC	0.518

L* = Lightness; a* = redness/greenness; b* = yellowness/blueness.

## Supplementary Materials

The following are available online at https://www.mdpi.com/2076-3921/8/10/442/s1, Figure S1: Chromatogram of *O. Africana* acetone leaves extract, Figure S2: Chromatogram of *O. Africana* ethanolic leaves extract; Table S1: Correlation between physicochemical and sensory measured variables for beef burger added of OA leaves aqueous extract (Aq) during storage at +4 °C; Table S2: Correlation between physicochemical and sensory measured variables for beef burger added of OA leaves acetone extract (Ac) during storage at +4 °C; Table S3: Correlation between physicochemical and sensory measured variables for beef burger added of OA leaves ethanol extract (Et) during storage at +4 °C; Table S4: Correlation between physicochemical and sensory measured variables for beef burger added of commercial mix ((+) control) during storage at +4 °C; Table S5: Correlation between physicochemical and sensory measured variables for beef burger during storage at +4 °C ((-) Control).
