# Supplementary material for: Phytochemical Constituents and Antioxidant Activity of Oudneya Africana L. Leaves Extracts: Evaluation Effects on Fatty Acids and Proteins Oxidation of Beef Burger during Refrigerated Storage
Source: Antioxidants (Basel). 2019 Oct 1;8(10):442. doi: 10.3390/antiox8100442 (PMC6826378; doi:10.3390/antiox8100442)
Supplement: Supplementary file 1 [file antioxidants-08-00442-s001.pdf]

Table S1. Correlation between physicochemical and sensory measured variables for beef burger added of OA leaves aqueous extract (Aq) during storage at +4°C.

**Aq**	PH	L	a	b	TBARS	Thiol	HO	RO	AO	PO	EO	MC	FC
PH	1												
L	-0,433	1											
a	-0,913**	0,141	1										
b	-0,320	0,082	0,571	1									
TBARS	0,111	-0,517	0,164	0,393	1								
Thiol	-0,852**	0,408	0,741**	0,259	-0,029	1							
HO	-0,410	-0,586	0,655*	0,252	0,379	-0,029	1						
RO	0,829**	-0,180	-0,785**	-0,205	0,184	0,379	-0,521	1					
AO	0,834**	-0,248	-0,763**	-0,155	0,154	0,184	-0,439	0,928**	1				
PO	0,833**	-0,221	-0,855**	-0,420	-0,122	0,154	-0,423	0,820**	0,884**	1			
EO	-0,877**	0,489	0,801**	0,305	-0,352	-0,122	0,332	-0,686*	-0,647*	-0,637*	1		
MC	-0,676*	0,159	0,830**	0,412	0,120	-0,352	0,545	-0,586*	-0,482	-0,511	0,693*	1	
FC	-0,882**	-0,153	0,905**	0,393	-0,096	0,120	0,805**	-0,811**	-0,743**	-0,788**	0,824**	0,807**	1

Table S2. Correlation between physicochemical and sensory measured variables for beef burger added of OA leaves acetone extract (Ac) during storage at +4 °C.

**Ac**	**PH**	**L**	**a**	**b**	**TBARS**	**Thiol**	**HO**	**RO**	**AO**	**PO**	**EO**	**MC**	**FC**
**PH**	1												
**L**	0,247	1											
**a**	-0,791**	0,221	1										
**b**	-0,259	0,621*	0,743**	1									
**TBARS**	-0,749**	-0,183	0,802**	0,606*	1								
**Thiol**	-0,658*	0,070	0,398	0,094	0,223	1							
**HO**	-0,783**	0,259	0,903**	0,636*	0,723**	0,428	1						
**RO**	0,942**	0,117	-0,849**	-0,390	-0,763**	-0,611*	-0,830**	1					
**AO**	0,934**	0,047	-0,855**	-0,463	-0,773**	-0,670*	-0,795**	0,955**	1				
**PO**	0,900**	0,193	-0,801**	-0,303	-0,772**	-0,491	-0,766**	0,892**	0,864**	1			
**EO**	-0,735**	-0,249	0,795**	0,548	0,948**	0,162	0,713**	-0,749**	-0,711**	-0,723**	1		
**MC**	-0,617*	-0,034	0,828**	0,682*	0,830**	0,124	0,687*	-0,607*	-0,624*	-0,571	0,906**	1	
**FC**	-0,679*	0,052	0,745**	0,612*	0,695*	, 0541	0,665*	-0,742**	-0,777**	-0,483	0,688*	0,736**	1

Table S3. Correlation between physicochemical and sensory measured variables for beef burger added of OA leaves ethanol extract (Et) during storage at +4°C.

Et	PH	L	a	b	TBARS	Thiol	HO	RO	AO	PO	EO	MC	FC
**PH**	1												
**L**	-0,893**	1											
**a**	-0,630*	0,395	1										
**b**	-0,748**	0,579*	0,972**	1									
**TBARS**	-0,882**	0,797**	0,739**	0,835**	1								
**Thiol**	-0,669*	0,742**	0,213	0,355	0,624*	1							
**HO**	-0,514	0,341	0,543	0,600*	0,556	0,395	1						
**RO**	0,831**	-0,570	-0,587*	-0,612*	-0,642*	-0,367	-0,353	1					
**AO**	0,813**	-0,662*	-0,587*	-0,630*	-0,794**	-0,645*	-0,252	0,817**	1				
**PO**	0,968**	-0,839**	-0,574	-0,680*	-0,867**	-0,641*	-0,488	0,783**	0,783**	1			
**EO**	-0,450	0,215	0,167	0,170	0,487	0,159	0,238	-0,564	-0,492	-0,534	1		
**MC**	-0,465	0,110	0,571	0,495	0,387	0,060	0,417	-0,667*	-0,582*	-0,475	0,452	1	
**FC**	-0,814**	0,574	0,756**	0,773**	0,803**	0,329	0,454	-0,786**	-0,786**	-0,812**	0,588*	0,807**	1

Table S4. Correlation between physicochemical and sensory measured variables for beef burger added of commercial mix (control +) during storage at +4°C.

Control +	PH	L	a	b	TBARS	Thiol	HO	RO	AO	PO	EO	MC	FC
**PH**	1												
**L**	-0,373	1											
**a**	-0,864**	0,519	1										
**b**	-0,777**	0,557	0,977**	1									
**TBARS**	-0,847**	0,503	0,986**	0,971**	1								
**Thiol**	-0,713**	0,088	0,296	0,201	0,295	1							
**HO**	-0,756**	0,221	0,832**	0,795**	0,881**	0,339	1						
**RO**	0,953**	-0,529	-0,864**	-0,808**	-0,846**	-0,654*	-0,746**	1					
**AO**	0,924**	-0,557	-0,883**	-0,814**	-0,848**	-0,543	-0,673*	0,902**	1				
**PO**	0,952**	-0,532	-0,867**	-0,809**	-0,846**	0,654*	-0,746**	0,976**	0,959**	1			
**EO**	-0,628*	-0,098	0,638*	0,518	0,627*	0,221	0,650*	-0,496	-0,472	-0,451	1		
**MC**	-0,563	-0,105	0,601*	0,519	0,637*	0,188	0,752**	-0,488	-0,366	-0,416	0,929**	1	
**FC**	-0,807**	0,614*	0,860**	0,828**	0,888**	0,356	0,717**	-0,839**	-0,851**	-0,839**	0,491	0,526	1

Table S5. Correlation between physicochemical and sensory measured variables for beef burger during storage at +4°C (Control -).

Control -	PH	L	a	b	TBARS	Thiol	HO	RO	AO	PO	EO	MC	FC
PH	1												
L	-0,309	1											
a	-0,499	-0,183	1										
b	0,371	-0,128	0,545	1									
TBARS	0,812**	-0,148	-0,749**	-0,097	1								
Thiol	-0,538	0,327	0,194	-0,164	-0,501	1							
HO	-0,720**	0,205	0,413	-0,170	-0,841**	0,606*	1						
RO	0,959**	-0,270	-0,500	0,359	0,729**	-0,512	-0,633*	1					
AO	0,960**	-0,319	-0,519	0,309	0,789**	-0,539	-0,716**	0,977**	1				
PO	0,967**	-0,207	-0,488	0,405	0,734**	-0,502	-0,639*	0,979**	0,937**	1			
EO	-0,405	-0,224	0,186	-0,335	-0,334	-0,093	0,539	-0,371	-0,404	-0,364	1		
MC	-0,755**	0,300	0,414	-0,204	-0,744**	0,343	0,720**	-0,762**	-0,759**	-0,788**	0,246	1	
FC	-0,819**	0,326	0,444	-0,244	-0,729**	0,337	0,652*	-0,821**	-0,799**	-0,861**	0,215	0,970**	1

Significance levels: * P ≤ 0.05, ** P ≤ 0.01
